# Real-time dual-modal photoacoustic and fluorescence small animal imaging

**DOI:** 10.1016/j.pacs.2024.100593

**Published:** 2024-02-02

**Authors:** Yu Sun, Yibing Wang, Wenzhao Li, Changhui Li

**Affiliations:** a Department of Biomedical Engineering, School of Future Technology, Peking University, Beijing 100871, China; b National Biomedical Imaging Center, Peking University, Beijing 100871, China

**Keywords:** Real time, Small animal imaging, Fluorescence imaging, Photoacoustic imaging, Multimodal imaging

## Abstract

By combining optical absorption contrast and acoustic resolution, photoacoustic imaging (PAI) has broken the barrier in depth for high-resolution optical imaging. Meanwhile, Fluorescence imaging (FLI), owing to advantages of high sensitivity and high specificity with abundant fluorescence agents and proteins, has always been playing a key role in live animal studies. Based on different optical contrast mechanisms, PAI and FLI can provide important complementary information to each other. In this work, we uniquely designed a Photoacoustic-Fluorescence (PA-FL) imaging system that provides real-time dual modality imaging, in which a half-ring ultrasonic array is employed for high quality PA tomography and a specially designed optical window allows simultaneous whole-body fluorescence imaging. The performance of this dual modality system was demonstrated in live animal studies, including real-time monitoring of perfusion and metabolic processes of fluorescent dyes. Our study indicates that the PA-FL imaging system has unique potential for live small animal research.

## 1 Introduction

Small animal models, especially rodents, play an important role for life science and pre-clinical research. Many noninvasive imaging technologies have been developed for small animal research. Among various imaging methods, the live animal fluorescence imaging (FLI) is mostly widely employed owing to its superior advantages, including non-invasive and non-ionizing mechanisms, high sensitivity and specificity, abundant molecular tracers, as well as real-time imaging over large field of view (FOV) [1], [2], [3], [4]. However, the strong tissue light scattering substantially limits the image resolution for FLI in deep tissues. Over the past decade, the photoacoustic imaging (PAI), which uniquely combines optical absorption contrast and ultrasonic detection[5], becomes a powerful method for live animal imaging with high resolution at unprecedented depth[6], [7], [8], [9], [10], [11], [12], [13], [14], [15], [16], [17].

Besides its superior in structural and functional imaging of blood vessels, many more PA contrast agents and molecular tracers are being intensively explored. Notably, most of these agents are poor fluorophores [18]. Therefore, based on different imaging contrast, imaging depth and spatial resolution, PAI and FLI can provide important complementary information to each other. Therefore, it is of great value to explore the integration of PAI with FLI for live animal studies. Several studies have reported their efforts to combine PAI results with FLI results for small animal research[19], [20], [21], [22]. These integrated systems either performed PAI and FLI in a sequential way or take long time to finish dual modal imaging, which are suitable for monitoring relatively slow changes. However, the real-time synchronization for PAI and FLI is also important. It is because not only the body motion due to heart beating and breathing could affect the spatial registration of these two modalities, but also there are transient physiological processes, such as intravenous drug delivery or perfusion in kidney [23], [24], all of which demands real-time synchronous imaging. Here, we report a real-time PA-FL dual modality imaging method, which is equipped with an ultrasonic array to provide real-time PAI two-dimension (2D) imaging, and the simultaneous whole-body FLI is acquired through an optical window in the PAI system. We successfully performed in vivo real-time imaging of mice, presenting the fluorescence dyes perfusion and metabolic process not only over the whole-body by FLI but also at unprecedented depth and resolution by PAI. Our results demonstrate the system has great potential for small animal studies, including drug delivery[25], dynamic metabolic molecular tracing, and monitoring other fast physiological or pathological processes.

## 2 System setup

### 2.1 Dual-modal imaging system

**Fig. 1:**
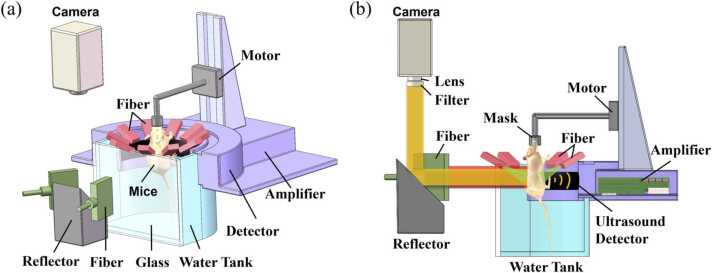
Dual-modal imaging system. (a) 3D schematic diagram of the system; (b) Illustration of both light and sound path ways.

The PAI-FLI system consists of two subsystems: FLI subsystem and PAI subsystem. As shown in Fig. 1, The PAI system has a customized 256-element half-ring ultrasonic transducer array (by ULSO TECH Inc, China), with central frequency of 5.0 MHz and one-way bandwidth of 80%. The half-ring has a diameter of 100 mm, and each array element is cylindrically focused in the elevational direction with a focal length of 40 mm. To maximize the signal to noise ratio (SNR), a self-developed 256-channel preamplifier (40 dB gain) is directly connected with the ultrasonic transducer array. Then, the amplified PA signals are parallelly received by a 256-channel data acquisition (DAQ) instrument (Marsonics: DAQ, Tsingpai Tech-Co, China, 6 dB gain) at 40 MHz sampling rate. The PA signal is excited by an Optical Parametric Oscillator (OPO) pulsed laser (Innolas SpitLight 600, Germany) with a repetition rate of 10 Hz. The laser is coupled into a 1-to-10 fiber bundle, and each branch end has a rectangle shape of 1 × 7 mm. The branch ends are evenly distributed around the imaged target, forming a nearly uniform circular illumination pattern, as shown in Fig. 1. A water tank is used for coupling ultrasound (US) signal to the half-ring detector, and the animal body were immersed into water during imaging with its head out of water attaching to a gas mask.

In order to simultaneously perform whole-body FLI, the water tank has an optical transparent window in the opposite of the US array, allowing both FL excitation and emission acquisition, as shown in Fig. 1. A 1-to-2 fiber bundle is used to excite FL signal via illuminating through the optical window, and the emitted FL signal was reflected by a tilted mirror (45 degrees) into a FL camera (Hamamatsu Flash 4.0, Japan). A florescence filter is mounted before the camera’s lens. In the following experiment, a 785 nm continuous laser (CNI MDL-III-785, China) was used to excite FL signal.

To synchronize PAI and FLI, when the OPO laser emits a laser pulsed, a photodetector (Thorlabs DET100A2) detects the laser pulse and generate a synchronizing trigger signal to both DAQ and FL camera to acquire data. To avoid possible photons from strong pulsed laser into the camera, we set a 10-ms delay time for camera to start expose. Camera exposure time set to 70 ms. During the imaging process, the small animal is vertically fixed on an animal holder into the water tank. The water temperature is maintained at 36 °C, and an electrical translational stage can move the animal body up and down to obtain photoacoustic images at arbitrary body slice. During in vivo experiments, an air mask covers the animal nose with 1.5% vaporized isoflurane gas.

### 2.2 System characteristics

**Fig. 2:**
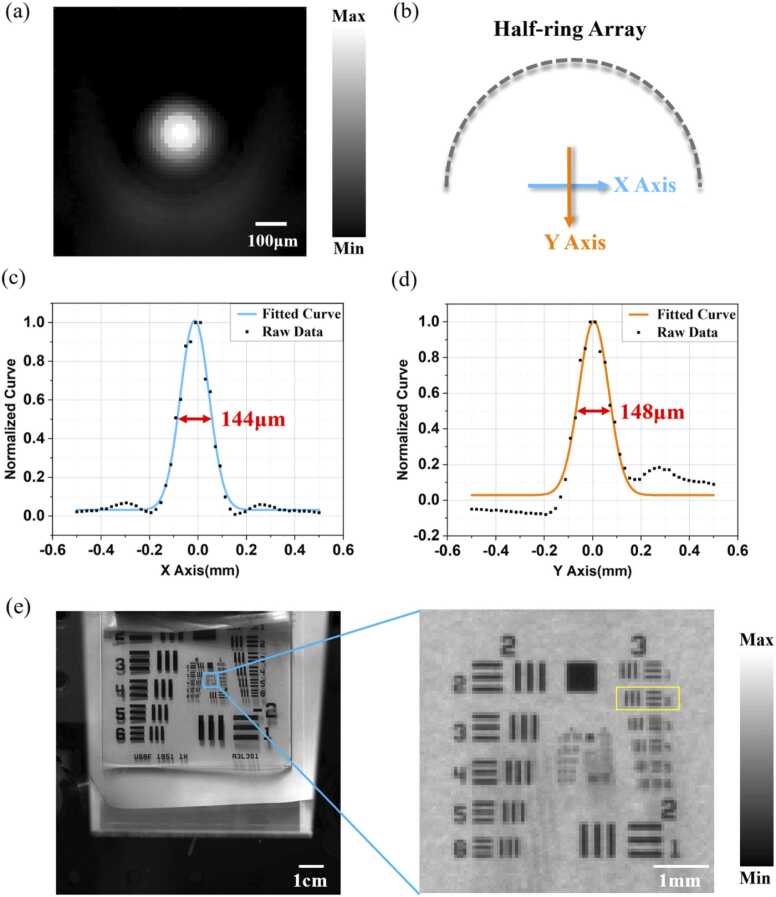
PAI-FI system resolution testing results. (a) Photoacoustic image of a human hair; (b) Schematic diagram of X and Y axis; (c) X-direction PA resolution; (d) Y-direction PA resolution;(e) Camera resolution chart under fluorescence imaging system;.

We used a human hair with the diameter of ∼90 µm for PAI system resolution testing. The hairline was placed vertically at the center of the half-ring ultrasound array and the measured resolution at the center point is ∼150 µm, as shown in Fig. 2(a-d). The PAI image reconstruction is accelerated by a customized GPU algorithm, achieving a reconstruction time less than 30 ms (GPU: RTX3060 Ti). Therefore, the PA imaging can be displayed in real time. Regarding the Fluorescence Imaging (FLI) setup, we employed a scientific CMOS camera (Hamamatsu Flash 4.0, Japan), equipped with a sensor resolution of 2048 by 2048 pixels. The camera, featuring a lens with a focal length of 55 cm, provides a field of view (FOV) of approximately 11 cm by 11 cm. This FOV is sufficient to cover the whole body of a small animal. For resolution assessment, as depicted in Fig. 2(e), we used FLI to image a resolution chart (Thorlabs R3L3S1P) that was placed at the intended location to fix the animal. The highest distinguishable pair of horizontal and vertical lines, as enclosed in the yellow box in Fig. 2(e), correspond to the second element of group three (8.98 line pairs per millimeter, lp/mm). This delineation indicates the maximal spatial resolution attainable by our fluorescence imaging system.

## 3 Imaging results

### 3.1 In vivo photoacoustic imaging

**Fig. 3:**
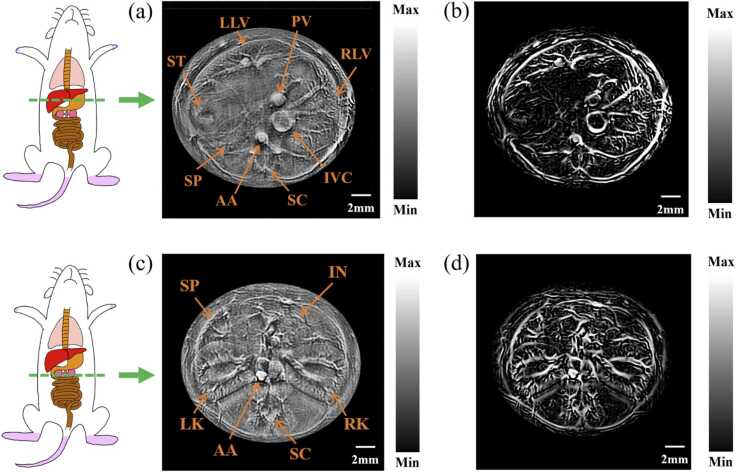
Photoacoustic imaging of mouse body. (a) PA reconstruction imaging of mouse liver;(b) Photoacoustic imaging of mouse liver after Frangi vascular filtering; (c)Photoacoustic reconstruction imaging of mouse kidneys;(d) Photoacoustic imaging of mouse kidneys after Frangi vascular filtering; AA, abdominal aorta; In, intestinal tract; IVC, inferior vena cava; LK, left kidney; LLV, left lobe of liver; PV, portal vein; RK, right kidney; RLV, right lobe of liver; SC, spinal cord; SP, spleen; St, stomach.

To demonstrate the PAI imaging performance, we used the 1064 nm laser to excite PA signal. A ∼20 g nude Balb/C mouse was used, which had a trunk diameter of approximately 16 mm. The total energy illuminated onto the body surface is 36 mJ, and the calculated fluency is 12 mJ/cm^2^, which is far less than the ANSI safety limit (100 mJ/cm^2^ at 1064 nm)[5]. Fig. 3 shows two cross-sectional (B-scan) PA imaging results, which clearly show the structure of multiple organs and tissues in this mouse, including abdominal aorta, intestinal tract, inferior vena cava, left and kidneys, left and right lobes of liver, portal vein, spinal cord, spleen and stomach. Besides the original reconstructed results, we also showed results after Frangi vascular filtering to enhance the display of vascular networks. From the imaging results, it can be seen that our half-ring PAI system has excellent imaging capability for small animal bodies.

### 3.2 Dual-modal in vivo real-time imaging of fast dye perfusion process

**Fig. 4:**
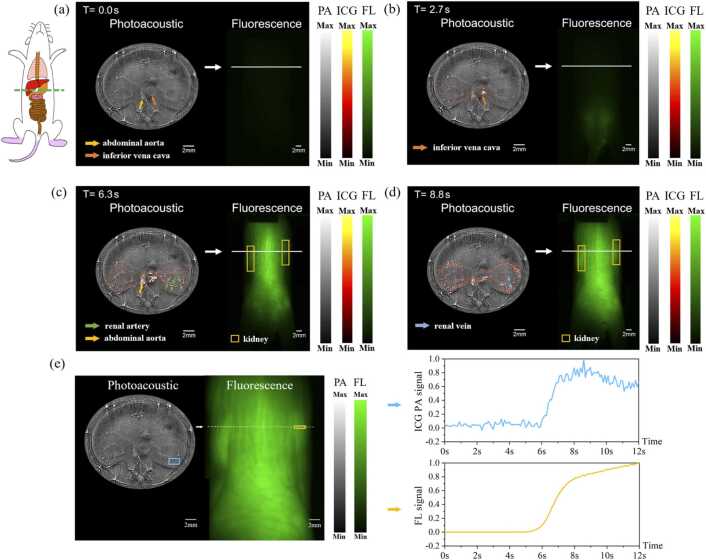
Fluorescence and photoacoustic images at different times in renal perfusion experiments. (a-d) After injection of ICG into the tail vein, photoacoustic and fluorescence images at t = 0.0 s, 2.7 s, 6.3 s, 8.8 s. The left side is the photoacoustic image, the right side is the fluorescence image. (e)The average photoacoustic signal of indocyanine green (ICG) within the region outlined by the blue box showed a change over time, as did the average fluorescence signal within the region designated by the yellow box.

For this study. We injected the indocyanine green (ICG) dye into a mouse via tail vein injection. It is known that ICG does not goes through renal metabolism, it only pass the kidney vasculature quickly via blood circulation. During the entire ICG perfusion process, the dual-modal system simultaneously performs real-time synchronous FL and PA imaging, as shown in Fig. 4. We used red pseudo color to indicate the dynamic ICG signal overlapping on PA structural grayscale imaging. The dynamic ICG signal is the result of subtraction of photoacoustic images, obtained by subtracting the baseline photoacoustic image (acquired before ICG injection) from each registered photoacoustic image (obtained after ICG injection).

Fig. 4 shows the fluorescence and photoacoustic images collected at different times within 10 s after the start of tail vein injection. The photoacoustic (PA) image is displayed on the left, with the corresponding fluorescence (FL) image shown on the right. We adjusted the vertical position to make PA imaging show the cross section along the kidney location, as indicated by the white line overlapped on FLI results, while the FLI shows whole-body imaging. As shown in Fig. 4(c-d), the yellow boxes in the right fluorescent image correspond to the positions of the left and right kidneys, respectively. According to PAI results, ICG dye flowed passing the inferior vena cava at about 2.7 s toward the heart, and flowed into the kidney from the thoracic aorta in about 6.3 s, then ICG dye perfused into the entire kidney vascular network in about 8.2 s. Evidenced by the data depicted in Fig. 4(e), there is a simultaneous enhancement of the fluorescence signal intensity and the photoacoustic signal intensity of ICG at the location of the corresponding kidney. The signal-to-noise ratio (SNR) for the ICG photoacoustic signal is measured at 27 dB and the SNR for the fluorescence signal is 64 dB. The above results indicate that PA image demonstrates superior spatial resolution within the cross-sectional area of the kidney and the FL image offers whole-body images concurrently and exhibits heightened sensitivity. It is worth noting that at the moment of 2.7 s, although strong PA signal indicating the arrival of ICG to the inferior vena cava, there is very weak FL signal due to strong tissue light scattering that substantially attenuated both FL excitation and emission photons. More results were provided in the online animation movie (Media 1).

Supplementary material related to this article can be found online at doi:10.1016/j.pacs.2024.100593.

The following is the Supplementary material related to this article Media 1..

Media 1

### 3.3 Dual-modal in vivo imaging of dye metabolism

In this study, we continued to monitor the metabolism process of ICG as a demonstration. For a healthy mouse, the injected ICG will goes through liver to gallbladder, and then enter intestines to be expelled out of body. In this study, we injected a certain concentration of ICG solution through the tail vein and monitored the signal changes in the liver and intestines within one hour after injection.

**Fig. 5:**
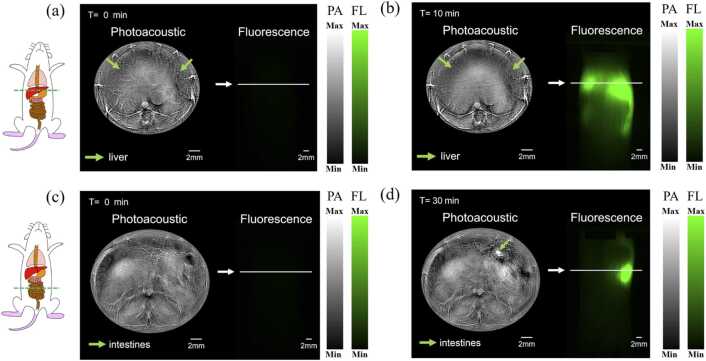
Photoacoustic and fluorescence images at different times in ICG metabolic experiments (a) Photoacoustic and fluorescence image in liver before ICG injection; (b) PA and FL image of the liver 10 min after ICG injection; (c) Photoacoustic and fluorescence image in intestines before ICG injection; (d) PA and FL image in intestines 30 min after ICG injection.

Since we need to monitor several locations, a motorized translational stage was used to change the mouse’s vertical position, and PAI scanned the mouse body at a step size of 0.1 mm over 30 mm, costing about 60 s to finish one cycle. Fig. 5 demonstrated two typical cross sections for liver and intestine, and simultaneous whole-body FLI was performed side by side. After injection, much of the ICG is absorbed by the liver within ∼10 min, which substantially blocked PA excitation light (790 nm) that lead an obviously decrease in image quality for deep organs. Fig. 5(c) is the result for 12.5 mm below the liver, which is intestine. It clearly shows that the much ICG reaches intestine within ∼30 min. The simultaneous FLI results shows the whole-body dynamics of FL signals, which is in overall consistent with PAI results. Our results are also consistent with the cognition of the metabolic pathway of ICG. More results are provided in online animation movie (Media 2–3). The above experiments prove that the system has the ability to observe the dynamic whole-body metabolic process of fluorescent dyes in small living animals.

Supplementary material related to this article can be found online at doi:10.1016/j.pacs.2024.100593.

The following is the Supplementary material related to this article Media 2..

Media S2

The following is the Supplementary material related to this article Media S3..

Media S3

## 4 Material and methods

### 4.1 Animal preparation and experiment

All experimental procedures were carried out in conformity with the laboratory animal protocols approved by the Animal Research Committee of Peking University. Nude mice aged 8 to 12 weeks (Balb/c nude, Charles river; ∼20 g body weight, male) were used for in vivo imaging and ICG experiments. The food supply was stopped 12 h before the experiment, but the water supply was maintained, which was used to empty the metabolites in the intestine. Before the experiment, depilatory cream was first used to remove the sparse hair of the mice, reducing the photoacoustic signal from the epidermis. Throughout the experiment, mouse was maintained under anesthesia with 1.5% vaporized isoflurane. The animal body was immersed in water tank filled with deionized water and maintain at around 36 °C. The motor controls the small animal holder to scan along the z-axis at a constant speed, enabling photoacoustic imaging at any position. In the body structural imaging experiment of Fig. 3, the laser wavelength was 1064 nm, the repetition rate was 10 Hz, and the illumination intensity of the laser was 12 mJ/cm^2^, which is far below the ANSI laser safety standard limit. In the ICG experiment, the wavelength of the laser used was 790 nm, the repetition rate was 10 Hz, and the illumination intensity of the laser was 8 mJ/cm^2^, which is also far below the ANSI laser safety standard limit. The ICG was injected via a self-made tail vein indwelling needle, which allows the injection of dyes while the animal body was immersed. The concentrations of ICG solution used in perfusion and metabolic path way experiments are 5 mg/ml (total 125 μg) and 0.3 mg/ml (total 30 μg), respectively.

### 4.2 Data processing and image reconstruction

For in vivo animal imaging, we used the dual-speed-sound back-projection algorithm implemented in MATLAB to reconstruct photoacoustic images[14], [26]. In vivo photoacoustic imaging, as shown in Fig. 3(a)(c),we processed photoacoustic images to improve the contrast through the following steps:(1) used a high-pass filter with a passband frequency of 0.8 MHz and a stopband frequency of 0.3 MHz to enhance vascular image. (2) Used contrast limited adaptive histogram equalization(CLAHE) to enhance contrast[27]. As shown in Fig. 3(b)(d), we applied a set of Hessian-based Frangi vascular filters to enhance the display of vascular networks[28]. The data processing steps are as follows:(1) Used a high-pass filter with a passband frequency of 0.8 MHz and a stopband frequency of 0.3 MHz to suppress the low-frequency. (2) Setting PA images negative values to zero, restricting all PA images values to the non-negative range. (3) Applying the Frangi filter to PA images that consist only of non-negative values.(4) Used contrast limited adaptive histogram equalization (CLAHE) after Frangi vascular filters.

In the dye metabolism experiment, only a high pass filter with a passband frequency of 0.1 MHz and a stopband frequency of 0.05 MHz is used to suppress the DC bias and low-frequency noise from the amplifier circuit. In the dye perfusion experiment, still use the above filter to filter low-frequency. We acquired photoacoustic imaging data both pre-injection and post-injection. The datasets were then aligned using the Demons registration algorithm [29], resulting in the grayscale photoacoustic images shown in Fig. 4. To obtain the dynamic indocyanine green (ICG) photoacoustic signals, we subtracted the baseline photoacoustic image (acquired before ICG injection) from each registered image obtained thereafter. The ICG photoacoustic images were subsequently processed with a temporal low-pass filter, which has a cutoff frequency of 1 Hz, in order to mitigate heartbeat-induced amplitude fluctuations in the photoacoustic signal. This filtering step is based on the understanding that the kinetics of ICG perfusion within the tissue are considerably slower than the rate of the mouse's cardiac cycle. To clearly demonstrate the renal perfusion process, only the dynamic ICG photoacoustic signals of the kidney and its surrounding major blood vessels are displayed as red pseudo colors in Fig. 4, while ignoring PA signal changes in other organs[23]. As shown in Fig. 4(e), the average photoacoustic signal of indocyanine green (ICG) within the region outlined by the blue box showed a change over time, as did the average fluorescence signal within the region designated by the yellow box. The size of the blue box is 2.0 mm × 1.0 mm and the size of the yellow box is 2.0 mm × 0.5 mm. These two regions correspond in spatial position.

### 4.3 Dual-mode synchronization scheme

**Fig. 6:**
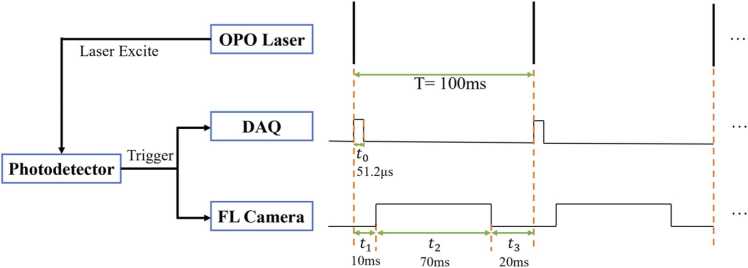
Schematic diagram of dual-mode synchronous operation.

We used a 10 Hz OPO laser with a repetition period of 100 ms to excite the PA signal, while a 785 nm CW laser was used to excite FL signal. When a photodetector detects the pulse laser from the OPO laser, it emits a pulse signal to trigger both the fluorescence camera and the Data Acquisition System (DAQ). Once the trigger is received, the PA's DAQ system captures 2048 data points at 40 MHz, completing in 51.2 μs. However, the fluorescent camera will delay for 10 ms to start exposure, which is to avoid the fluorescence imaging being influenced by the intense OPO laser light. The camera’s exposure time is set to 70 ms, as shown in Fig. 6.

## 5 Discussion and Conclusion

In this work, we developed a real-time PA-FL dual-modality imaging system, employing a half-ring ultrasonic array that enables real-time 2D PAI, in conjunction with whole-body FLI through an optical window. This novel imaging system successfully achieved in vivo real-time imaging of the perfusion and metabolic processes of fluorescent dyes in mice. The system integrates the advantages of FLI and PAI, in which FLI has a real-time large FOV of whole-body coverage and high sensitivity, and PAI offers high resolution in deep tissue imaging and provides tissue structural information. The real-time synchronous imaging abilities of the dual-modality fluorescence and photoacoustic system unlock novel avenues for small animal research and preclinical studies, such as monitoring the whole-body dynamic drug delivery, tracing cancer cells that are labeled by dual-modal molecular tracers, studying neurovascular coupling in both central and peripheral nervous systems.

In future work, multi-wavelength PAI will be implemented to provide more functional and structural information, including oxygen saturation and tissue composition. Besides system upgrade, we will also optimize reconstruction algorithms to alleviate artifacts caused by the limited view of the half-ring configuration, including advanced iterative reconstruction methods and artificial intelligent aided methods. In conclusion, our work provides an unique real-time dual-modality system that integrates two powerful small animal imaging methods, photoacoustic imaging and fluorescence imaging, providing a platform to perform various dynamic molecular and functional imaging studies.

## CRediT authorship contribution statement

**Changhui Li**: Writing – review & editing, Supervision, Resources, Project administration, Methodology, Investigation, Funding acquisition, Conceptualization. **Sun Yu**: Writing – original draft, Methodology, System design and realization, Animal experiments, Data reconstruction and process. **Yibing Wang**: Real-time reconstruction programming. **Wenzhao Li**: Electrical technical support and realization.

## Declaration of Competing Interest

We declare that we have no financial and personal relationships with other people or organizations that can inappropriately influence our work. there is no professional or other personal interest of any nature or kind in any product, service and/or company that could be construed as influencing the position presented in, or the review of, the manuscript entitled “Real-time dual-modal photoacoustic and fluorescence small animal imaging system”.

## Data Availability

Data will be made available on request.
